# Maoto, a Traditional Japanese Herbal Medicine, Inhibits Uncoating of Influenza Virus

**DOI:** 10.1155/2017/1062065

**Published:** 2017-08-22

**Authors:** Shinta Masui, Shigeki Nabeshima, Kazuhiko Ajisaka, Kei Yamauchi, Ryota Itoh, Kazunari Ishii, Toshinori Soejima, Kenji Hiromatsu

**Affiliations:** ^1^General Medicine, Fukuoka University Hospital, Fukuoka, Japan; ^2^Department of Microbiology and Immunology, Fukuoka University School of Medicine, 7-45-1 Nanakuma, Jonan-ku, Fukuoka 814-0180, Japan

## Abstract

We previously reported in randomized controlled trials that maoto, a traditional herbal medicine, showed clinical and virological efficacy for seasonal influenza. In this study, a culturing system for influenza was used to test the effect of maoto. A549 cells in the culture were infected with influenza virus A (PR8) and followed after treatment with maoto; the virus titers in the culture supernatant, intracellular viral proteins, and viral RNA were determined. When infected cells were cultured with maoto for 24 hr, the virus titer and protein were significantly reduced compared with medium only. Other subtypes, A/H3N2, H1N1pdm, and B, were also inhibited by maoto. Proliferation of viral RNA in a 6 hr culture was inhibited by maoto in the early phase, especially in the first 30 min. Focusing on the entry step of the influenza virus, we found that endosomal pH, regulated by vacuolar-type H^+^ ATPase (V-ATPase) located in the membrane, was increased when treated with maoto. We also found that uncoating of influenza viruses was also inhibited by maoto, resulting in the increase of the number of virus particles in endosomes. These results strongly suggest that the inhibition of endosomal acidification by maoto results in blocking influenza virus entry to cytoplasm, probably through the inhibition of V-ATPase. The present study provides evidence that supports the clinical use of maoto for the treatment of influenza.

## 1. Introduction

Influenza remains an important infectious disease that causes major pandemic outbreaks worldwide. The currently available anti-influenza drugs, the M2 proton channel inhibitors amantadine and rimantadine and the neuraminidase inhibitors oseltamivir, zanamivir, and laninamivir, have made a remarkable contribution to the treatment of influenza [1]. Neuraminidase inhibitors have also been effective for patients infected with pandemic influenza 2009 [2, 3]. However, adverse effects, compliance problems, limited supply, and high cost are major concerns when prescribing neuraminidase inhibitors [4]. In addition, the future efficacy of these antivirals may be limited as stable, transmissible drug-resistant strains emerge, as happened with A/H1N1pdm [5–8]. Attention to new therapeutics for the treatment of influenza continues to be important.

Traditional herbal medicines have long played an important role in countries in the Far East, especially Japan, China, and Korea. Maoto (Ma-Huang-Tang in Chinese) is traditionally prescribed for acute febrile diseases or upper respiratory tract infection [9, 10]. We and others previously reported in nonrandomized [11, 12] and randomized controlled trials [13, 14] that maoto has clinical efficacy for seasonal influenza, without severe adverse effects. In our study of adult patients randomly assigned maoto granules, oseltamivir, or zanamivir, analysis was done of the duration of fever, total symptom score, and viral shedding. The results showed that maoto was well tolerated and that it had equivalent clinical and virological efficacy to oseltamivir and zanamivir in a group of healthy adults with seasonal influenza [13]. Our results were supported by a recent study of experimental infection with influenza virus in mice, in which the viral titer was decreased in the nasal and bronchoalveolar lavage fluids of mice treated with maoto [15].

In spite of its clinical advantages, the anti-influenza mechanism of maoto remains unclear. We studied the anti-influenza effect of maoto using a culturing system in which lung epithelial cells were experimentally infected with influenza virus in the presence of maoto. After attachment to cell surface receptors, influenza viruses are trapped by the endosome, where the envelope of the influenza virus fuses with the endosome membrane in an acidified condition; then nucleocapsids are released into the cytosol. The intraluminal space of the endosome and lysosome is acidified by vacuolar-type H^+^ ATPase (V-ATPase) at the endosomal membrane. We examined whether or not the function of V-ATPase at the endosomal membrane was inhibited by maoto, with a quick increase of endosomal pH possibly resulting in the influenza virus being unable to enter the cytoplasm. We show here evidence that maoto has an anti-influenza drug effect on the host's endocytic system and defense.

## 2. Materials and Methods

### 2.1. Cells and Viruses

The following cell lines were used for experimental infection: human lung carcinoma cell line A549, human keratinocyte cell line HaCat, human cervix carcinoma cell line HeLa, and mouse embryonic fibroblast cell line MEF. Madin-Darby canine kidney (MDCK) cells were used for the titration of infective influenza virus in the culture supernatant. For the main experiments the Puerto Rico/8/34 (PR8) influenza A virus strain was used, and for other experiments A/Victoria/210/2009 (H3N2), A/California/7/2009 (H1N1, pdm09), and B/Brisbane/60/2008 were used. The latter three strains were the components of the influenza vaccine used in the 2011-2012 winter season in Japan. PR8 was provided by Chemo-Sero-Therapeutic Research Institute (Kumamoto, Japan), and all other viruses were provided by the National Institute of Infectious Diseases (Tokyo, Japan). The virus titer in supernatant was determined on MDCK cells according to the methods of Reed and Muench [16], using a 50% tissue culture infectious dose (TCID50).

### 2.2. Infection with Influenza Virus

For the attachment of virus to the cell surface, suspended virus at a moi of 1–100 was added to confluent monolayers of A549 cells in 12-well tissue culture plates for 1 hr, on ice. Cells were washed twice with cold PBS to remove the extracellular viruses and then cultured for the indicated amount of time with or without antivirus reagents at 37°C.

### 2.3. Antivirus Reagents

Maoto is a multicomponent formulation extracted from four plants: Ephedra Herb, Apricot Kernel, Cinnamon Bark, and Glycyrrhiza Root (Table 1). Maoto powder for experimental use, provided by Tsumura & Co (Tokyo, Japan), was made from the extracts obtained through decoction by boiling in water, concentration, and spray drying. Maoto powder was dissolved and incubated in warm EMEM for 1 hr. Supernatant was collected after sedimentation at 3,000 ×g, filtered through a 0.45 *µ*M filter, and stored at −80°C until use. The concentration of maoto used for the culture was 400 *µ*g/ml. Amantadine, the M2-channel inhibitor used, was purchased from Sigma-Aldrich, and laninamivir, the neuraminidase inhibitor used, was provided by Daiichi Sankyo (Tokyo, Japan) [17].

### 2.4. Immunofluorescence Microscopy

To visualize the influenza protein, the cells were stained with anti-matrix protein 2 (M2) (Thermo Scientific, Hudson, NH), anti-haemagglutinin (HA) (Santa Cruz Biotechnology, Dallas, TX), or anti-nucleoprotein (NP) (ViroStat, Portland, ME) mAb and visualized by FITC-conjugated secondary mAb followed by staining with DAPI nucleic acid stain (invitrogen) for 1 min; then the cover glasses were mounted on microscope slides. The cells were analyzed on a Keyence all-in-one fluorescence microscope BZ-9000 with a 60x lens (Osaka, Japan) and confocal microscopy (Zeiss LSM710, Oberkochen, Germany). Acidified organelles were stained with dyes, LysoSensor green (ThermoFisher, Yokohama, Japan) and Acridine orange (ThermoFisher), and were assayed by confocal microscopy (Zeiss LSM710, Oberkochen, Germany).

### 2.5. Flow Cytometric Analysis

Cultured cells were collected and stained with anti-M2 (Thermo Scientific) mAb for 1 hr, washed with PBS, and stained with FITC-conjugated mouse mAb (Invitrogen). After the cell wash, cells were gated in forward and side scatter using flow cytometry, FACSCanto (BD, Franklin Lakes, NJ): up to 30,000 gated cells were acquired for the analysis. For the toxicity assay of maoto, the cells were stained with 7-amino-actinomycin D and Annexin V.

### 2.6. PCR Analysis

RNA extraction was carried out using ISOGEN II (Nippon Gene, Tokyo, Japan) and cDNA synthesis with a prime script RT reagent kit with gDNA eraser (Takara, Tokyo, Japan), according to the manufacturer's instructions.

Real-time PCR analysis was performed using a 7500 real-time PCR system (Applied Biosystems, Foster City, CA) with SYBR Green Kit (Takara). The following primers were used: GAPDH-Fw: 5′-TCCACCACCCTGTTCCTGTA-3′, GAPDH-Rv: 5′-ACCACAGTCCATGCCATCAC-3′, PA-Fw: 5′-GCTTCTTATCGTTCAGGCTCTTAGG-3′, PA-Rv: 5′-CCGAGAAGCATTAAGCAAAACCCAG-3′, NS-Fw: 5′-CAGGACATACTGATGAGGATG-3′, NS-Rv: 5′-GTTTCAGAGACTCGAACTGTG-3′, MxA-Fw: 5′-TTCAGCACCTGATGGCCTATC-3′, MxA-Rv: 5′-TGGATGATCAAAGGGATGTGG-3′, PKR-Fw: 5′-TCTCTGGCGGTCTTCAGAAT-3′, PKR-Rv: 5′-ACTCCCTGCTTCTGACGGTA-3′, IFN-beta-Fw: 5′-TGCTCTCCTGTTGTGCTTCTCC-3′, IFN-beta-Rv: 5′-CATCTCATAGATGGTCAATGCGG-3′, IFN-lambda1-Fw: 5′-GAAGCAGTTGCGATTTAGCC-3′, and IFN-lambda1-Rv: 5′-GAAGCTCGCTAGCTCCTGTG-3′.

### 2.7. Statistical Analysis

All samples were done at least in triplicate, with each experiment repeated at least twice. Results are expressed as means ± standard deviation. Differences between groups were determined using the unpaired Student *t*-test. *p* values of < 0.05 were considered statistically significant.

## 3. Results

### 3.1. Toxicity of Maoto to Cells and Viruses

**Figure 1:**
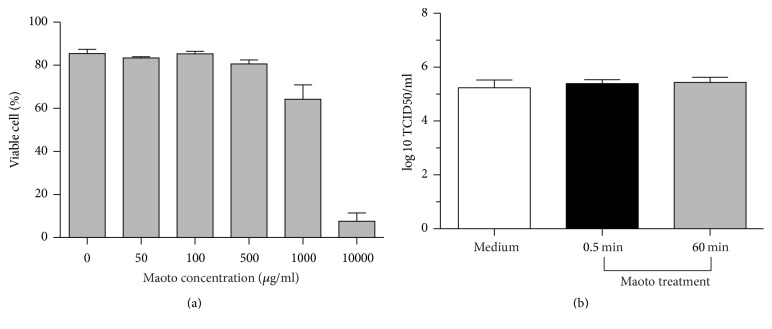
Maoto is not toxic for cell lines and influenza viruses. (a) A549 cells were cultured with 0 to 1,000 *µ*g/ml maoto for 24 hr and then assayed by flow cytometry after cell staining with 7-amino-actinomycin D and Annexin V, from which the double negative population is determined as viable cells. (b) A dense concentration of PR8 (10^9^ TCID50/ml) was incubated with medium only, or pretreated with 400 *µ*g/ml maoto for 0.5 min or 60 min, and was used to infect A549 cells (moi = 1) followed by the culture for 24 hr. Culture supernatants were assayed for the titer of infectious virus.

The cytotoxic effect of maoto was first assayed by flow cytometry after cell staining with 7-amino-actinomycin D and Annexin V in which the double negative population is determined as viable cells. When A549 cells were cultured with 0 to 1,000 *µ*g/ml maoto for 24 hr, 80% or more cells were viable by flow cytometry (Figure 1(a)). The concentration of maoto in the following experiments was 400 *µ*g/ml. The direct effect of maoto on virions was also examined. A dense concentration of PR8 (10^9^ TCID50/ml) was incubated with medium only or pretreated with 400 *µ*g/ml maoto for 0.5 min or 60 min and then was used to infect A549 cells (moi = 1) followed by the culturing (maoto concentration < 1 *µ*g/ml). Twenty-four hours later, no difference was found in the virus titer between cells infected with PR8 pretreated with maoto for 0.5 min or 60 min, demonstrating no direct effect of maoto on virions (Figure 1(b)).

### 3.2. In Vitro Anti-Influenza Virus Effect of Maoto on Virus Titer

**Figure 2:**
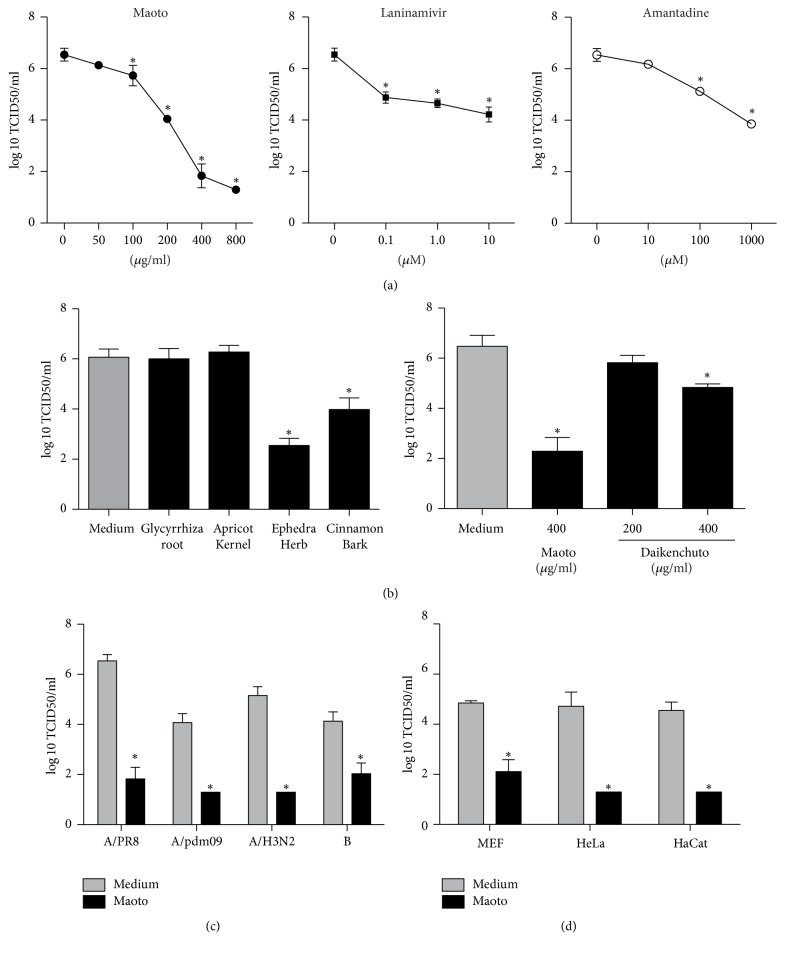
Maoto inhibited influenza virus propagation in vitro. All cell lines infected with influenza viruses (moi = 1) were cultured for 24 hr; then culture supernatants were assayed for the titer of infectious virus. (a) A549 cells infected with PR8 were cultured with various concentrations of maoto, laninamivir, or amantadine. (b) Left: A549 cells infected with PR8 were cultured with the components of maoto, Ephedra Herb (130 *µ*g/ml), Apricot Kernel (130 *µ*g/ml), Cinnamon Bark (100 *µ*g/ml), or Glycyrrhiza Root (40 *µ*g/ml), according to their weight ratios in maoto (Table 1). Right: A549 cells treated with the control, daikenchuto, another Kampo medicine, were infected with PR8 (moi = 1). (c) A549 cells infected with A/California/7/2009 (H1N1, pdm09), A/Victoria/210/2009 (H3N2), or B/Brisbane/60/2008 were cultured with or without maoto. (d) MEF, HeLa, or HaCat cell lines infected with PR8 were cultured with or without maoto. ^*∗*^*p* < 0.05 versus medium only.

In order to investigate the anti-influenza virus effect of maoto, we cultured A549 cells infected with PR8 in various concentrations of maoto, laninamivir, or amantadine, and the culture supernatant was assayed for the titer of extracellular infectious virus. The virus titer without antivirus reagent was 6.54 log_10_⁡ TCID_50_/ml. When maoto was added, the virus titer was significantly reduced in a dose-dependent manner (Figure 2(a)). The lowest virus titer, 1.3 log_10_⁡ TCID_50_/ml, was obtained with 800 *µ*g/ml maoto. Laninamivir and amantadine also reduced the virus titer in a dose-dependent manner; however, the antivirus effect of 400 and 800 *µ*g/ml maoto significantly exceeded that of 10 *µ*M laninamivir and 1,000 *µ*M amantadine. Because maoto is extracted from four plants, we next analyzed which plants were responsible for the inhibitory effect on the virus titer (Figure 2(b), left). Only Ephedra Herb and Cinnamon Bark had a significant antivirus effect. A control test of daikenchuto, another Kampo medicine used for the treatment of abdominal pain, showed that it was also effective, but the effect was limited compared to maoto (Figure 2(b), right).

To generalize this inhibitory effect of maoto on the virus titer, experiments were performed using other subtypes of influenza virus and different cell lines. When A549 cells were infected with A/California/7/2009 (H1N1, pdm09), A/Victoria/210/2009 (H3N2), or B/Brisbane/60/2008, the virus titer in wells cultured with maoto was significantly lower than the titers with the control medium (Figure 2(c)). Similarly, when PR8 was used to infect MEF, HeLa, or HaCat cells, maoto also had lower viral titers than with the medium only (Figure 2(d)). These findings demonstrate that maoto had superior capacity to inhibit the propagation of influenza virus compared to the tested neuraminidase and M2 proton channel inhibitors and that the effect is consistent for diverse subtypes (A/H1N1, A/H3N2, and B) of influenza virus.

### 3.3. In Vitro Effect of Maoto on Intracellular Influenza Protein

**Figure 3:**
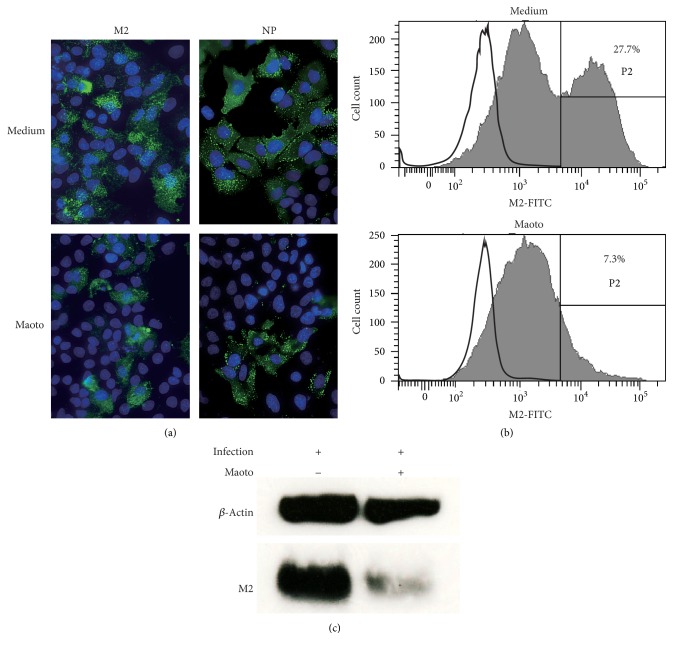
Maoto inhibits intracellular protein derived from PR8. A549 cells infected with PR8 (moi = 1) were cultured with or without maoto for 24 hr. Proteins derived from PR8 were visualized by labeled mAb. (a) Cells were stained with mAb to M2 or NP (green) and assayed by fluorescence microscopy. Nuclei were dyed blue. (b) Cells were stained with mAb to M2, and fluorescent intensity was assayed by flow cytometry. (c) Cell lysates were assayed by western blot analysis for M2 protein.

To visualize the components of the influenza virus, cells were cultured for 24 hr in the presence of maoto, stained with mAb to M2 or NP of PR8, and imaged by immunofluorescence microscopy (Figure 3(a)). The number of maoto-treated M2- and NP-positive cells was reduced when compared with the control. The fluorescence dots of M2 or NP per cell were weaker in specimen from the culture with maoto than from that of the control medium. This finding was confirmed by flow cytometric analysis (Figure 2(b)). M2-positive cells from the cultures with the control medium and maoto were 27.7% and 7.3%, respectively. Furthermore, in the M2-positive area, the fluorescence intensity of each cell treated with maoto was much weaker than that of cells of the control medium, showing that influenza virus components, such as M2, were fewer in each of the cells treated with maoto. Finally, western blot analysis showed that cell lysates from the culture treated with maoto contained less M2 protein than the control medium (Figure 3(c)). These observations suggest that the anti-influenza virus effect of maoto depends not only on the reduced frequency of cells infected with influenza virus, but also on reduced production of influenza virus per cell.

### 3.4. Minimal Requirement of Interferons, PKR, and MxA on Antiviral Effect

**Figure 4:**
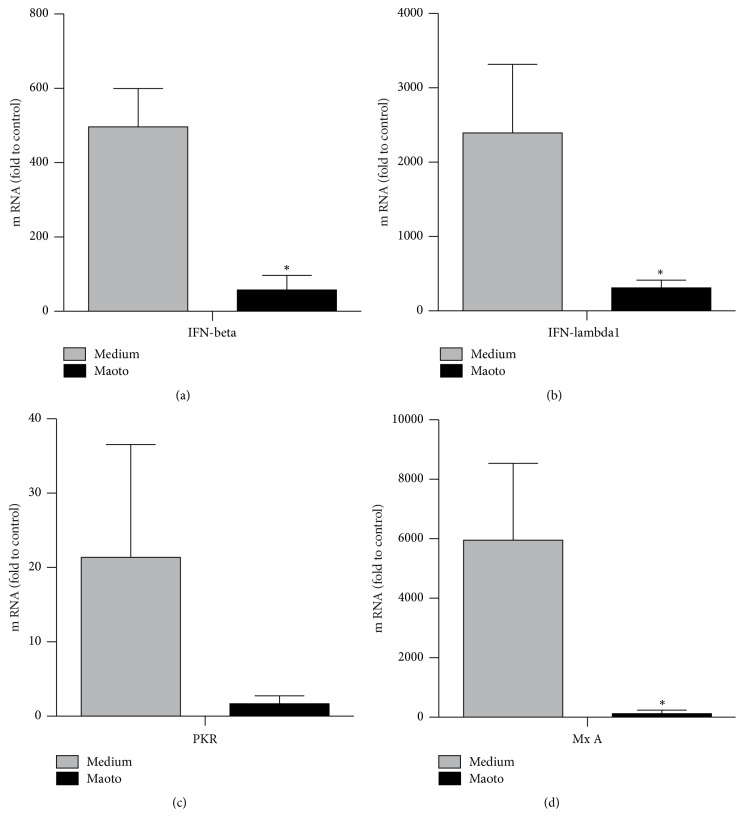
Maoto did not induce an intracellular antiviral molecule related to type I or type III IFNs in influenza virus infection. A549 cells infected with PR8 were cultured with or without maoto, and mRNA levels of IFN-*β*, IFN-*λ*1, PKR, and MxA were measured by real-time PCR. ^*∗*^*p* < 0.05 versus medium only.

In influenza virus infection, type I interferons (IFN *α* and *β*) and type III interferons (IFN-*λ*1 to IFN-*λ*3) contribute to innate antiviral responses through IFN-stimulated antiviral molecules, such as myxovirus resistance gene A (MxA), RNA-dependent protein kinase (PKR), and oligoadenylate synthetase (OAS) [18–20]. As a possible mechanism of the anti-influenza virus effect, we first hypothesized that maoto might induce IFNs and IFN-induced antivirus molecules. We therefore examined the possibility that maoto influenced the induction of IFN-*β*, IFN-*λ*1, PKR, or MxA. Twenty-four hours later, mRNA levels of IFN-*β*, IFN-*λ*1, PKR, and MxA were measured by real-time PCR (Figure 4). As compared with medium only, the mRNA levels of IFN-*β*, IFN-*λ*1, PKR, and MxA cultured with maoto were remarkably reduced, which suggests that the anti-influenza effect of maoto did not result from the induction of the type I and III IFNs systems.

### 3.5. Inhibition of Influenza Virus Entry by Maoto

**Figure 5:**
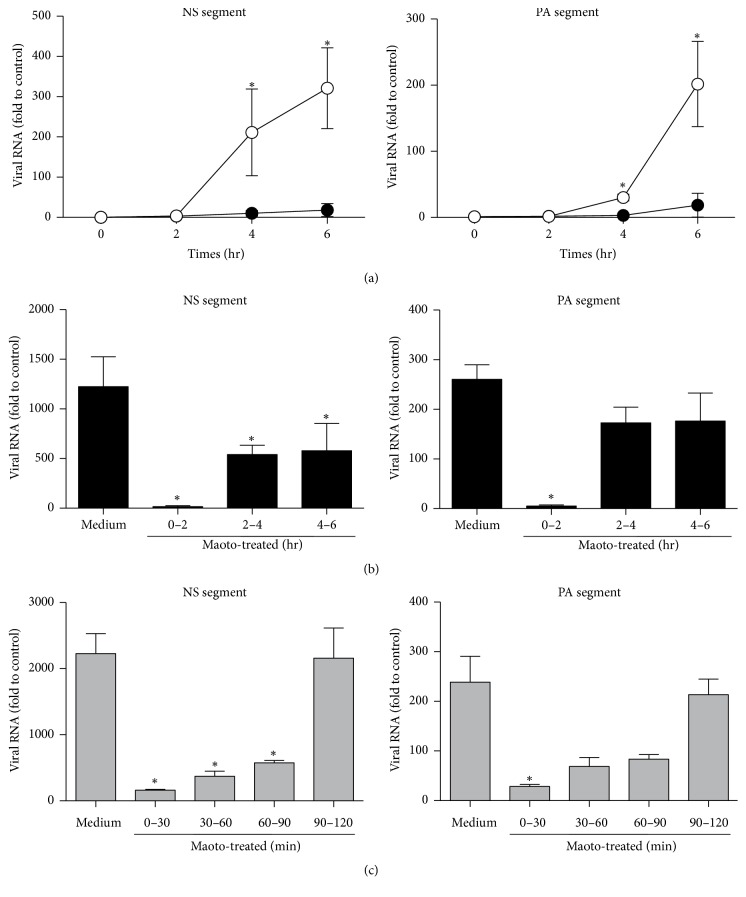
The effect of maoto was observed in the very early phase of influenza virus infection. A549 cells infected with PR8 (moi = 1) were cultured for 6 hr. The length of maoto treatment was varied in each experiment. Levels of mRNA for NS and PA segment of PR8 were assayed by real-time PCR analysis. (a) Cells were treated with maoto for 6 hr. Open and closed circles indicate control and maoto, respectively. (b) Maoto treatment for 0–2, 2–4, or 4–6 hr. (c) Maoto treatment for 0–30, 30–60, 60–90, or 90–120 min. ^*∗*^*p* < 0.05 versus medium only.

Second, we hypothesized that maoto blocked the entry step of influenza virus into the cytoplasm. Because the replication cycle of influenza virus needs 6–8 hrs from attachment to shedding, the assay used to determine virus entry into the cytoplasm of host cells requires short-term culturing to avoid the influence of progeny viruses. We next examined viral RNA replication (NS and PA segments) under the short-term culture condition, resulting in the viral RNA being remarkably inhibited by maoto throughout 6 hrs after infection (Figure 5(a)). To elucidate the timing at which maoto showed its antiviral effect, we divided the treatment time with maoto into two hour intervals: 0–2, 2–4, and 4–6 hrs of culturing time (Figure 5(b)). We found that viral RNA replication was remarkably inhibited in the 0–2 hr interval, indicating that protection from influenza virus by maoto mainly occurs in the early phase of infection. We further divided the first 2 hrs of culturing into 30 min intervals and found that the earliest phase (0–30 min) had the most antiviral activity (Figure 5(c)). Because it has been reported that the influenza virus takes 20 min to be uncoated after attachment to the cell surface [21], these observations clearly show that maoto blocked the entry or the fusion step.

### 3.6. Maoto Inhibits Endosomal Acidification and Uncoating of Influenza Virus

**Figure 6:**
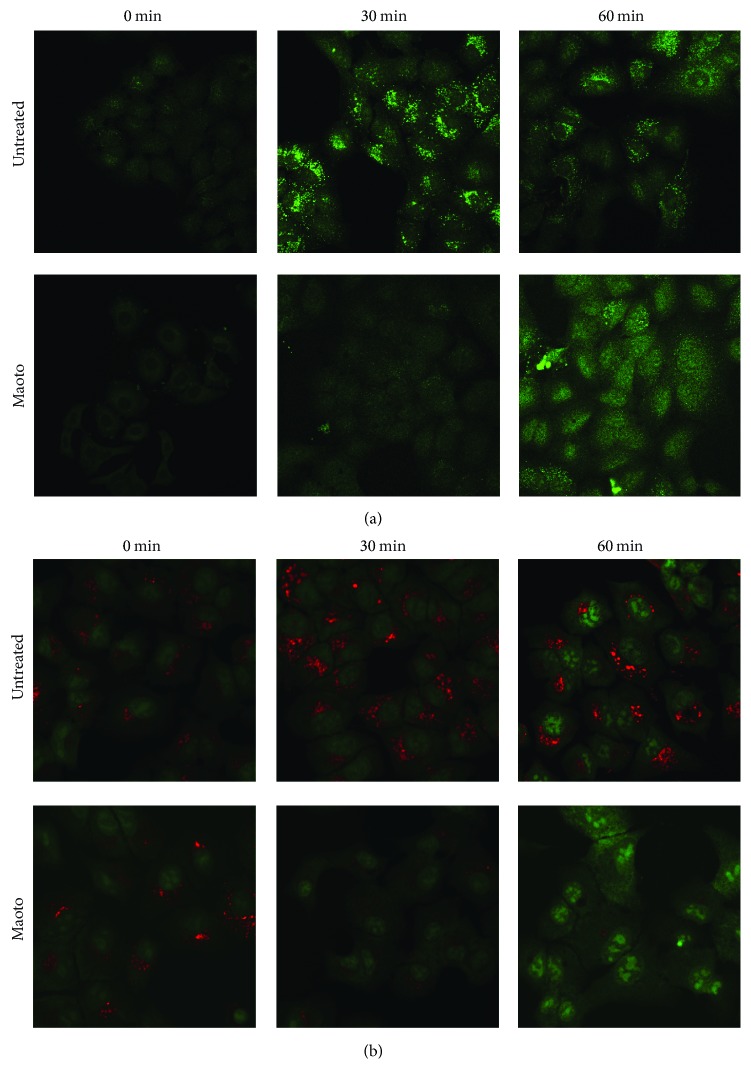
Endosomal acidification was inhibited by maoto. A549 cells were incubated with PR8 for 1 hr at 4°C (moi = 1), washed by PBS, and then cultured at 37°C with or without maoto in the presence of LysoSensor green (a) or Acridine orange (b). Acidified endosomes and lysosomes at 0, 30, and 60 min were visualized by fluorescence microscopy. The former can be visualized as green fluorescence and the latter as orange fluorescence in high concentration whereas it was green in low concentration.

It has been reported that an acidic condition in endosomes is essential for the uncoating process of influenza virus infection and that it triggers viral envelope fusion activity [22]. We hypothesized that maoto inhibits endosomal acidification by blocking V-ATPase present at the membrane of intracellular organelles, such as endosomes and lysosomes. V-ATPase pumps the cytosolic H^+^ into their lumen in an ATP-dependent manner [23]. LysoSensor green and Acridine orange, weak base dyes, are selectively taken up by living cells and concentrated in acidic organelles (pH 4.5–6.0) such as endosomes and lysosomes. The former can be visualized as green fluorescence and the latter as orange fluorescence: orange in high concentration and green in low concentration. To determine if maoto inhibits endosomal acidification, we cultured PR8-infected A549 cells with or without maoto for 0, 30, and 60 min and then added LysoSensor green (Figure 6(a)) or Acridine orange (Figure 6(b)). In untreated cells, an accumulation of LysoSensor green and Acridine orange was seen in cytoplasm 30 min after infection, showing that acidified organelles, such as endosomes and lysosomes, were increased in number. After 60 min, dotted fluorescence was reduced and accumulated in the perinuclear region. On the other hand, at 30 min after infection maoto treatment had clearly reduced the accumulation of LysoSensor green and Acridine orange, suggesting that acidification of endosomes and lysosomes was inhibited. After 60 min, dotted LysoSensor green and Acridine orange were reduced and the fluorescence diffusely spread to cytoplasm and nucleus. These observations suggest that maoto can block the V-ATPase function and inhibit the entry of influenza virus into cytoplasm.

**Figure 7:**
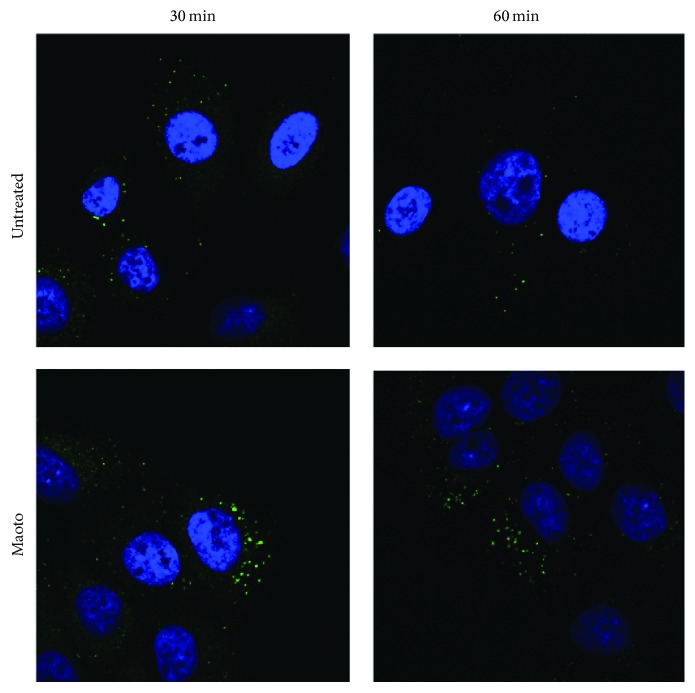
A549 cells were incubated with PR8 for 1 hr at 4°C (moi = 100), washed by PBS, and then cultured at 37°C with or without maoto. Intracellular influenza virus at 30 and 60 min after infection was visualized by the staining of mAb to HA (green) followed by staining with DAPI.

We further investigated whether or not influenza viruses could be uncoated, following the inhibition of endosomal acidification by maoto. The number of influenza virus particles visualized by HA-staining after treatment with maoto showed a greater increase when compared to the control (Figure 7), suggesting that uncoating of influenza virus was inhibited by maoto and that virus particles remained in endosomes.

## 4. Discussion

The present study demonstrates that maoto, a traditional Japanese herbal medicine, has the capacity to inhibit experimental infection with influenza virus PR8, as well as the subtypes A/H1N1(2009 pdm), A/H3N2, and B. Of the four plants included in maoto extracts, only Ephedra Herb and Cinnamon Bark had a significant antiviral effect. The antiviral effects of maoto were mainly explained by blocking the uncoating process of the influenza virus through the inhibition of V-ATPase, a proton pump located in the endosome and lysosome membranes.

Recently, several new reports related to Kampo and Chinese medicines for influenza infection have been focused on the molecular level [15, 24–28]. Known antivirals, such as neuraminidase inhibitors and M2 proton channel inhibitors, exert an antiviral effect by combining with the viral molecules and inhibiting their function. We did not show a direct effect of maoto on virions because our pretreatment with maoto for PR8 for 60 min did not reduce the viral titer (Figure 1). In a different way, maoto may have the potential to enhance the host's innate defense system and inhibit viral infection. Influenza virus infection induces proinflammatory cytokines, such as IL-1, IL-6, and type I IFNs, which play an important role in reducing the viral load. In the present study, after infection with influenza virus, cells treated with maoto produced less mRNA for type I IFNs and its downstream MxA and PKR. The possibilities are (1) that inhibited viral propagation by maoto might not induce the IFN system and (2) that maoto directly or indirectly inhibited the IFN system. Because epithelial cell lines such as the A549 used in the present study produce a lower amount of inflammatory cytokines, further experiments using plasmacytoid dendritic or macrophage cell lines will be necessary to clarify the effects of maoto on the cytokine system.

Our results correspond with a previous study showing an anti-influenza effect of Ephedra Herb, one of the plants included in maoto extracts, through the inhibition of endosomal acidification in vitro [29]. The present study did further experiments, including viral RNA replication in the early stage of infection, viral protein synthesis, induction of intracellular antiviral molecules, direct effect of maoto on virions, and the effect of maoto on other subtypes of influenza virus and cells, which can confirm and generalize the antiviral effect of maoto. It has been reported that bafilomycin A1, chloroquine, and diphyllin also block endosomal acidification and inhibit influenza virus propagation, although none of them are clinically used for influenza [30–32].

V-ATPase is a proton pump that acidifies the lumen of endomembrane organelles, such as lysosomes, endosomes, Golgi apparatus, and secretory granules and that is required for the entry of influenza virus into cells [33]. Influenza viruses attached to sialic acid on cell surface are transferred by coated vesicles to endosomes, where the luminal condition is kept to be acidified by V-ATPase present at the membrane. In the acidified condition, the envelope of the influenza virus can fuse with the endosomal membrane, which is followed by the release of nucleocapsids to cytosol. Recently, it has been reported that influenza viruses enhance the function of V-ATPase for their infectivity [34, 35]. V-ATPase activity was elevated during infection of influenza virus through a mechanism mediated by extracellular signal-regulated kinase (ERK) and phosphatidylinositol 3-kinase (PI3K) [36]. Maoto, as well as bafilomycin A1, may have an effect on V-ATPase or V-ATPase-related signaling systems, although the precise mechanism is unknown at present. We plan to do future study on the mechanism(s) of maoto on V-ATPase.

Maoto extracts consist of four plants and include hundreds of molecules [11, 15]. Inhibition of endosomal acidification is the major anti-influenza mechanism, but other mechanisms must be considered. The cinnamaldehyde derived from Cinnamon Bark, which is included in maoto, was reported to inhibit the growth of influenza virus through the a defect of translation [27]. Several Kampo medicines including maoto have been reported to inhibit the polymerase acidic protein of influenza A virus [24]. We previously reported, in a clinical trial, that maoto had a quick effect on influenza-related symptoms, such as fever, headache, and myalgia/arthralgia [12]. These effects may be explained by the inhibition of prostaglandin E2 and proinflammatory cytokines. Proinflammatory cytokines induce systemic inflammatory responses, which sometimes lead to harmful effects in the host, such as influenza-related acute respiratory distress syndrome, and encephalopathy [37, 38]. We think that maoto plays an important role in controlling both the viral load and inflammation.

Antiviral drugs mainly target viral molecules, such as neuraminidase and M2. These drugs are at risk of losing effectiveness by viral mutation. Kampo medicine targets the host cellular processes and enhances the defense system against various microorganisms, which can reduce the risk of viral mutation. In addition, maoto may have an effect on other viruses that use endosomes in the infection process, such as coronavirus, adenovirus, and togavirus. Interestingly, maoto has traditionally been used not only for influenza, but also for other acute febrile diseases. Our results confirm the common wisdom built over hundreds of years that the use of maoto is effective in the treatment of acute febrile diseases caused by various pathogens.

## Figures and Tables

**Table 1:** Maoto extract.

Plants	Weight ratio (%)	Major components
Ephedra Herb	32.3	Ephedrine, Tannin
Apricot Kernel	32.3	Amygdalin
Cinnamon Bark	25.8	Cinnamic aldehyde, Tannin
Glycyrrhiza Root	9.6	Glycyrrhizin
